# *Thelazia callipaeda* Eyeworms in American Black Bear, Pennsylvania, USA, 2023

**DOI:** 10.3201/eid3009.240679

**Published:** 2024-09

**Authors:** Caroline Sobotyk, Jaclyn Dietrich, Guilherme G. Verocai, Lauren Maxwell, Kevin Niedringhaus

**Affiliations:** University of Pennsylvania School of Veterinary Medicine, Philadelphia, Pennsylvania, USA (C. Sobotyk, J. Dietrich, L. Maxwell, K. Niedringhaus);; Texas A&M University College of Veterinary Medicine and Biomedical Sciences, College Station, Texas, USA (Guilherme G. Verocai)

**Keywords:** *Thelazia callipaeda*, eyeworm, emerging disease, thelaziosis, wildlife, parasites, zoonoses, Pennsylvania, United States

## Abstract

We identified a *Thelazia callipaeda* eyeworm in an American black bear in Pennsylvania, USA, on the basis of its morphological features and molecular analysis. Our finding highlights emergence of a *T. callipaeda* worm sylvatic transmission cycle in the United States.

Thelaziosis is an emerging zoonotic disease caused by nematodes of the genus *Thelazia* (Spirurida, Thelazioidea). In the United States, 3 zoonotic species have been identified: *Thelazia gulosa* (*1*), *T. californiensis* (*2*), and most recently *T. callipaeda* (*3*). In Asia and Europe, *T. callipaeda* is considered the main agent of thelaziosis in humans, domestic animals, and wild animals (*4*). Over the past decade, the geographic distribution and prevalence of *T. callipaeda* infection has increased worldwide in scale and intensity (*4*). The first autochthonous case in the United States was reported in 2018 in a domestic dog (*Canis lupus familiaris*) from New York with a history of unilateral epiphora and blepharospasm. Since then, additional cases in domestic dogs and cats have been reported, predominately from the northeastern United States (*3*,*5*).

*T. callipaeda* eyeworms are found in the conjunctival sac and lacrimal duct of the definitive host. They are transmitted when a male zoophilic secretophagous *Phortica variegata* fly ingests first-stage larvae from the host’s lachrymal secretions. In the vector, the first-stage larvae molt to the infective third-stage larvae in the testes, migrate to the mouthparts, and are transferred to another host during subsequent feeding on lachrymal secretions (*4*).

The role of wildlife in the epidemiology and emergence of *T. callipaeda* eyeworms is not completely known. In Europe, cases of *T. callipaeda* eyeworm infection have been detected in a wide range of hosts, including wild carnivores, omnivores, and lagomorphs (*6*,*7*). Wild canids, particularly red foxes (*Vulpes vulpes*), seem to play a large role in maintaining the sylvatic cycle in thelaziosis-endemic areas of Europe (*7*). However, knowledge of the sylvatic transmission cycle of *T. callipaeda* eyeworms, along with their environmental and anthropogenic factors, remains limited. Considering the emergence of those zoonotic nematodes in non–thelaziosis-endemic areas and the need for more information about their ecology and epidemiology in the United States, we report a case of *T. callipaeda* eyeworm infection in an American black bear (*Ursus americanus*) and identify a new geographic location of transmission.

## The Study

In November 2023, an adult, female American black bear was legally harvested in Coolbaugh Township, Monroe County, Pennsylvania. During processing of the bear for taxidermy preparation, multiple linear nematodes were observed behind the third eyelid. Nematodes were extracted and submitted for identification. Two additional harvested bears from Monroe and Pike Counties, Pennsylvania, were also reported to have similar ocular nematode infections, but specimens from those bears were not collected.

**Figure 1:**
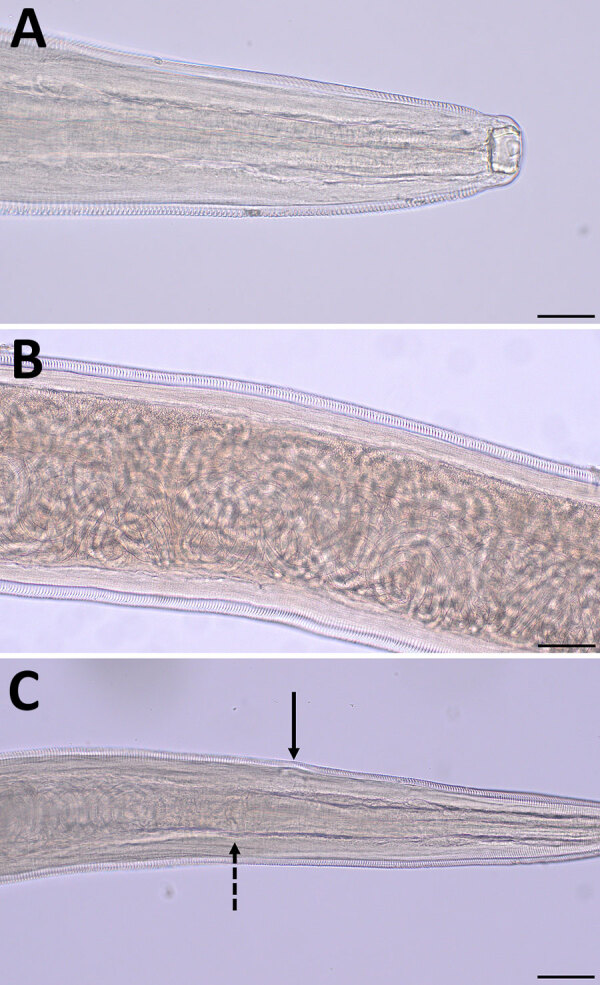
Morphologic features of adult female *Thelazia callipaeda* eyeworm isolated from an American black bear in Coolbaugh Township, Monroe County, Pennsylvania, USA, 2023. A) Anterior end showing the large, deep, cup‐shaped buccal cavity. Scale bar indicates 50 μm. B) Midbody region showing the thin transverse cuticular striations pattern and numerous coiled first-stage larvae. Scale bar indicates 100 μm. C) Anterior end showing the location of the vulvar opening anterior to the esophageal-intestinal junction. Dashed black arrow indicates esophageal-intestinal junction; solid black arrow indicates the vulval opening. Scale bar indicates 100 μm.

We identified 9 female and 4 male adult nematodes from the bear as *T. callipaeda* on the basis of morphologic and morphometric features (*8*). The nematodes were characterized by the presence of a cup-shaped buccal capsule and cuticular transverse striations, as well as the location of the vulvar opening anterior to the esophageal-intestinal junction on the female worms (Figure 1). Female nematodes were 1.16–1.46 cm long and 0.36–0.42 mm wide; male worms were 0.82–1.06 cm long and 0.31–0.42 mm wide. The number of transverse cuticular striations ranged from 160 to 400/mm in the cephalic, midbody, and caudal regions.

**Figure 2:**
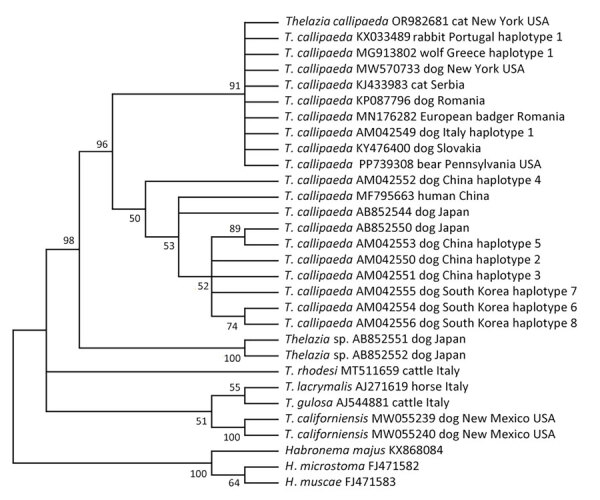
Phylogenetic relationship of *Thelazia callipaeda* isolate from an American black bear in Coolbaugh Township, Monroe County, Pennsylvania, USA, 2023 (GenBank accession no. PP739308), and other species of *Thelazia* available in GenBank (accession numbers shown). Analysis was performed by using the maximum-likelihood method (1,000 bootstrap replicates) in MEGA X version 11 (https://www.megasoftware.net). The best-fit nucleotide substitution model for the dataset was Tamura-Nei with a discrete gamma distribution, which was used to model evolutionary rate differences among sites (5 categories [+G, parameter = 0.2578]). That analysis involved 30 nucleotide sequences. There were 647 positions in the final dataset. Distances, defined as the number of nucleotide substitutions/site, were calculated by using that model. Branches corresponding to partitions reproduced in <50% of bootstrap replicates are collapsed.

We extracted genomic DNA from a midbody fragment of a female adult worm and amplified, sequenced, and analyzed the partial cytochrome oxidase c subunit I (*cox*1) gene, as previously described (*2*). We generated a 623-bp *cox*1 sequence (GenBank accession no. PP739308), which showed 99%–100% maximum identity with *T*. *callipaeda* sequences available in GenBank. Phylogenetic analysis was performed by using the maximum-likelihood method and confirmed the taxonomic identification of *T*. *callipaeda*. The isolate clustered with all previous isolates from domestic animals in North America and with some isolates from Europe (Figure 2), indicating circulation of the newly introduced pathogen in wildlife habitats and transmission from domestic animals to wildlife.

The presence of adult *T. callipaeda* eyeworms in an American black bear suggests the establishment of a sylvatic transmission cycle in the United States and expansion of the number of definitive host species used by the zoonotic nematode. In the past decade, wild carnivores have been identified as primary definitive hosts associated with the sylvatic cycle in thelaziosis- endemic and non–thelaziosis-endemic areas of Europe and Asia (*7*). American black bears are the most widely distributed species of bear in North America, inhabiting diverse regions throughout Mexico, Canada, and the United States (*9*). Given the bears’ extensive geographic distribution and frequent and close interaction with humans and pets (*10*), thelaziosis in the black bear population raises concerns about the rapidly increasing incidence and geographic range of *T. callipaeda* eyeworms in the United States. Although further research into the extent to which black bears play a role in the maintenance of the sylvatic cycle and transmission of *T. callipaeda* eyeworms is needed, the presence of the zoonotic nematode in such a wide range of hosts implicates exposure and risk for transmission to threatened and endangered species and direct or indirect risk for transmission to humans and domestic animals.
